# Regenerative Medicine of Epithelia: Lessons From the Past and Future Goals

**DOI:** 10.3389/fbioe.2021.652214

**Published:** 2021-03-25

**Authors:** Eleonora Maurizi, Davide Adamo, Federica Maria Magrelli, Giulia Galaverni, Eustachio Attico, Alessia Merra, Maria Benedetta Rizzarda Maffezzoni, Lorena Losi, Vincenzo Giuseppe Genna, Virginia Sceberras, Graziella Pellegrini

**Affiliations:** ^1^Holostem Terapie Avanzate S.r.l., Modena, Italy; ^2^Interdepartmental Centre for Regenerative Medicine “Stefano Ferrari”, University of Modena and Reggio Emilia, Modena, Italy; ^3^Department of Life Sciences, University of Modena and Reggio Emilia, Modena, Italy

**Keywords:** regenerative medicine, epithelia, clinical applications, cornea, oral mucosa, epidermolysis bullosa, trachea, salivary gland

## Abstract

This article explores examples of successful and unsuccessful regenerative medicine on human epithelia. To evaluate the applications of the first regenerated tissues, the analysis of the past successes and failures addresses some pending issues and lay the groundwork for developing new therapies. Research should still be encouraged to fill the gap between pathologies, clinical applications and what regenerative medicine can attain with current knowledge.

## Introduction

**FIGURE 1:**
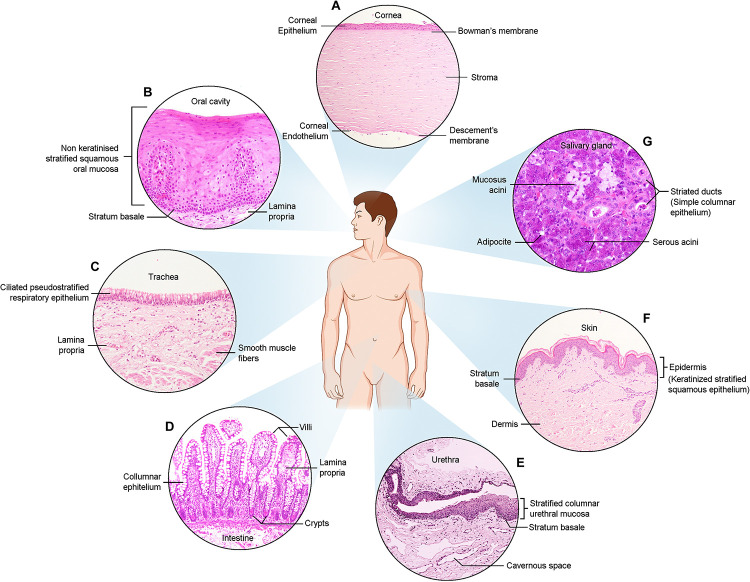
Haematoxylin and Eosin images of the described epithelia. **(A)** Cornea **(B)** Oral cavity **(C)** Trachea **(D)** Intestine **(E)** Urethra **(F)** Skin **(G)** Salivary gland.

Regeneration is often thought to belong to animal species phylogenetically distant from humans. However, human bodies are constantly rebuilding themselves, which means that we all have the chance to improve our health, if we can harness the regenerative capacity of our bodies. After the reconstitution of the hematopoietic system by bone marrow infusion (Thomas et al., 1957), the importance of stem cell content was elucidated by lengthy studies on clinical outcomes. Later on, brilliant scientists isolated stem cells capable to regenerate the human epidermis *in vitro* (Rheinwatd and Green, 1975) for subsequent transplantation to the human body, managing to heal very severe burns. This step marked the beginning of a new era, sketching out the idea that, by harnessing their power into the clinic, stem cells could be used to tackle a wide range of diseases. Indeed, many human tissues and organs possess the ability to self-renew due to specific stem cells, which generate progenitors, producing terminally differentiated cells. The cultures of these specific stem cells have been shown to fully restore some severely damaged tissues. Unfortunately, the clinical translation was delayed by many hurdles, as several human tissues do not regenerate, stem cells of some tissues have not been found yet, or we are still unable to stimulate them properly. A goal in regenerative medicine is to find ways to engineer tissues replacement and/or kick-start tissue regeneration in the body. The epithelia were the first cultured tissues because most of them are easily accessible and highly renewing. However, epithelia are highly specialized and some of them are characterized by various differentiation lineages, multiple signaling, and specific microenvironments on several matrices (Figure 1). Thus, their regeneration requires different and multidisciplinary medical expertise as they cover the whole body both internally and externally. To evaluate the applications of the first type of cultured stem cell in tissue regeneration, namely the epithelia, an analysis of the past clinical successes and failures can address some pending issues and support new applications.

## The Skin

The skin is the largest organ in our body and ensures not only a barrier against the environment and microorganisms but has also many other functions such as immune surveillance and tactile sensitivity. Its integrity is fundamental for survival; therefore, it renews constantly and comes with stem cells, similar to other lining epithelia. Its stem cells reside in the hair follicles, from where they can generate hair follicles, sebaceous glands, or the epidermis; however, some of them reside in the interfollicular epidermis.

The expression of several markers characterizes the interfollicular cells: cytokeratin (CK) 19 (Michel et al., 1996), β1-integrin (Jensen et al., 1999), α6-integrin (Li et al., 1998), desmoglein-3 (DSG3) (Wan et al., 2003) and high levels of melanoma chondroitin sulfate proteoglycan (MCSP) (Legg et al., 2003). Instead, C200 and CK15 identify hair follicle bulge cells (Ohyama et al., 2006; Inoue et al., 2009). However, these markers do not appear to be specific for the stem cells population. In 1999, the p63 transcription factor was essential in stratified epithelial regeneration (Yang et al., 1999). Two years later, Pellegrini et al. (2001) demonstrated that only small cells expressing high levels of p63 in the nuclei could be identified as epidermal and corneal stem cells in humans. Similarly, Yes-associated protein (YAP), a transcriptional activator involved in the Hippo pathway, was explicitly shown in the nuclei of human stem cells of the epidermis (De Rosa et al., 2019). A YAP target named survivin, more expressed in the hair follicles than in the interfollicular epidermis, is considered an additional stem cells marker (Marconi et al., 2007; Dallaglio et al., 2009).

The epidermal stem cell isolation occurred by chance, while Howard Green’s vision drove investigations into the discovery of a new outstanding technology: from a small skin biopsy, a large number of human epidermal keratinocytes were obtained by cultivating them on a feeder layer of lethally irradiated 3T3 cells (Rheinwatd and Green, 1975). This seminal study progressively defined a real microenvironment, providing adhesion, general and specific proliferative stimuli, as well as differentiation signals to obtain an appropriate polarization with the stratification of this vital tissue.

This technique has been considered the most successful approach compared with the cultivation of epidermal keratinocytes without feeder layer cells or on a feeder layer mitotically arrested by mitomycin C (Llames et al., 2015). The latter, for example, produced microchimerism in treated patients (Hultman et al., 1996), likely due to *in vivo* re-activation of temporarily arrested cells.

### Epidermal Stem Cells: The Leading Actors of Regeneration After Burns

In 1981, autologous cells grown from a small epidermal fragment to a graft were applied for the very first time in two patients, affected by third-degree burns (O’Connor et al., 1981).

The definitive proof of concept for autologous culture engraftment occurred in two children, who suffered burns covering over 97 and 98% of their body surfaces (Gallico et al., 1984). Both patients survived transplantation and died 20 years later due to complications unrelated to the burns.

The maintenance of stem cells in culture was investigated in 1987 by Barrandon and Green. These two scientists identified three types of clonogenic keratinocytes (holoclones, meroclones, and paraclones) by the clonal analysis of primary epidermal cultures (Barrandon and Green, 1987). Over the following years, this group and others demonstrated that the holoclones exhibit all stem cell features and are consequently able to generate meroclones (progenitor cells) and paraclones (transient amplifying cells) (Mathor et al., 1996; Pellegrini et al., 1999a; Larsson et al., 2014). Later on, after several clinical applications, De Luca et al. (2006) suggested that an adequate number of keratinocyte stem cells are necessary to produce a graft that can be successfully transplanted to patients and obtain long-term epidermal renewal.

The clinical outcomes of the first epidermis transplantations onto the muscular fascia were lifesaving in severe burns. However, the lack of dermis caused several drawbacks, such as scarring and wound contraction, indicating the need for connective tissue.

To address this issue, Cuono et al. (1987) and Chua et al. (2016) proposed a composite graft consisting of allogeneic skin as a source of dermis and autologous keratinocyte cultures to avoid the acute rejection caused by allogenic epidermis transplantation. This approach represents an important step forward in skin reconstruction (Cuono et al., 1987; Chua et al., 2016).

To overcome the lack of skin donors, many groups have studied different biological materials to reproduce the functions of connective tissue. Some examples are Matriderm or Integra—the most widely accepted artificial dermal substitutes—both made up of mainly bovine collagen (Chua et al., 2016; Girard et al., 2017).

Finally, Pellegrini et al. (1999b) and Ronfard et al. (2000) introduced modified fibrin glues as biodegradable carriers for cultured epidermis. This technology maintained the relative percentage of holoclones and meroclones, proving that fibrin does not induce clonal conversion and the consequent loss of epidermal stem cells. The engraftment of keratinocytes was high and permanent; and the presence of carriers prevented tissue shrinking, enabling a significant increase in the cultured tissue surface, with a reduction in time and cost. The introduction of re-absorbable carriers solved problems related to handling and transportation (Pellegrini et al., 1999b; Burd, 2000; Ronfard et al., 2000).

Alternative applications of autologous cultured keratinocyte sheets were produced as a wound dressing for treatment of superficial burns and chronic ulcers, without the need for engraftment, to promote wound healing (Johnsen et al., 2005).

### Skin Pigmentation Disorders: The Maintenance of a Correct Ratio Between Keratinocytes and Melanocytes

After the treatment of extensive and severe skin burns with epidermal keratinocytes, the skin discoloration drew attention to melanocytes contained in primary epidermal cultures, showing that they can grow in culture with keratinocytes. Melanocyte growth and differentiation are regulated by keratinocytes, and their contact is finely settled to maintain a physiological keratinocyte/melanocyte ratio (De Luca et al., 1988).

These findings laid the foundation for developing a new application for epidermal cultures, bearing a controlled number of melanocytes for treating patients affected by skin pigmentation disorders, such as piebaldism and some forms of vitiligo. The former occurs during embryological development, while the latter, a chronic inflammatory disease, affects approximately 0.5–1% of the global population (Singh et al., 2013). Both of these pathologies result in the absence of functional melanocytes, thus having strong impacts on patients’ quality of life and psychological well-being.

The first transplantation of cultured autologous melanocytes in a patient suffering from vitiligo was performed by Lerner et al. (1987) and then reproduced by several groups (Falabella et al., 1989; Plott et al., 1989; Kumagai and Uchikoshi, 1997). However, in 2000 Pellegrini’s group optimized the culture system, allowing normal and pathological melanocytes to proliferate in a constant ratio with respect to keratinocytes (Guerra et al., 2000). The cultured autografts were not only physiologically appropriate (with melanocytes in the basal layer, independent from the presence of dermis) but also functionally capable of developing dendritic arborizations with melanosome-containing processes and transferring melanosomes to the keratinocyte cytoplasm (De Luca et al., 1988).

The related treatment was effective in patients affected by stable vitiligo or piebaldism, where the healed epidermis was populated by the correct number of melanocytes, and stable repigmentation was obtained in approximately 40 patients (Guerra et al., 2000; Guerra et al., 2004).

Despite the proven efficacy, this treatment requires highly specialized personnel, as well as high manufacturing costs. To reduce costs, some other studies skipped the step of *in vitro* co-cultures, and cells were directly transplanted onto the depigmented area (Mulekar et al., 2008). A comparative study between autologous non-cultured epidermal cell suspension transplantation and its combination with non-cultured dermal cell suspension was carried out as a randomized clinical trial (NCT03013049) on 40 patients with stable vitiligo. This study suggested that the combined approach was more effective than the use of non-cultured epidermal cell suspension alone in patients with vitiligo stable from 3 to 6 months (Thakur et al., 2019). However, the efficacy of this novel approach has yet to be confirmed in the long term. Indeed, a large cohort of patients and a long follow-up are needed to confirm the stable repigmentation, as well as a comparative study between this approach and the transplantation of cultured epidermal autografts (Thakur et al., 2019).

### Achievements in Epidermolysis Bullosa: The Gene Therapy

The regenerative capacity of cultured epidermal keratinocytes leading to successful cell therapy approaches was further combined with gene therapy to treat genetic diseases affecting the skin. One devastating skin genetic disease is epidermolysis bullosa (EB), presenting with mucocutaneous blistering, erosions, ulceration, and fragility in response to minor trauma. It ranges from mild to severe until the point of being disabling or fatal due to the loss of function caused by the lack of protein adhesion (Bardhan et al., 2020).

Based on the ultrastructural sites of blister formation, mode of inheritance, genetics, and clinical phenotype, EB classification includes four major types: simplex (EBS), junctional (JEB), dystrophic (DEB), and kindler (KEB) (Fuchs, 1995; Mariath et al., 2020).

Morgan et al. (1987) first hypothesized that transduced epidermal cells could act as vehicles for the delivery of gene products through graft transplantation.

Thus, the first clinical confirmation of genetic correction in JEB came from Mavilio et al. (2006), where *ex vivo*-cultured keratinocytes derived from an adult patient affected by JEB were transduced with a retroviral vector expressing laminin-5 beta3 cDNA to produce an epidermal engineered graft. Impressively, this study showed that genetically corrected epidermal cells could regenerate an effective, functional skin. More than 6.5 years later, the follow-up of this patient showed a stable laminin 332 expression from the transgene (De Rosa et al., 2014), which is essential for mediating keratinocyte adhesion. Two further examples of effective skin restoration came from Bauer et al. (2017), who presented a successful gene therapy of two small skin areas of a 49-year-old woman and Hirsch et al. (2017), who showed the first regeneration of the entire human epidermis in a 7-year-old boy through autologous transgenic keratinocyte cultures. They proved that the transgenic regenerated epidermis was sustained only by a certain number of long-lived stem cells (holoclones), giving rise to a pool of short-lived progenitors, as widely proposed in different studies, such as those on corneal cells (Barrandon and Green, 1987; Pellegrini et al., 1999a; Rama et al., 2010).

These exciting advancements in combining cell therapy with gene therapy led to the regeneration of functional epithelial tissues and the treatment of diseases, such as EB, for which no effective therapy existed before. Further, they paved the way for the regeneration of other epithelial tissues in the human body.

## The Cornea

The experience gained from cell therapy for skin regeneration has led to the development of other stem cell-based treatments, such as corneal epithelium replacement, aimed at the restoration of visual acuity (Pellegrini et al., 1997; Rama et al., 2010).

The cornea is a unique avascular tissue that functions as a refractive component of the ocular surface system. It is composed of epithelium, stroma, and endothelium (DelMonte and Kim, 2011), and its main features, namely its mechanical strength and transparency, are prerequisites that allow vision. In fact, corneal trauma and burn (among others), causing the loss of corneal transparency and the consequent corneal opacity, represents the fourth leading cause of blindness in the world (Pascolini and Mariotti, 2012).

### Corneal Epithelial Repair and Regeneration Approaches

The discovery of corneal regeneration driven by the centripetal migration of cells from the limbus (between the central cornea and the adjacent conjunctiva) led to the identification of limbal stem cells (LSCs), which are responsible for corneal renewal.

When LSCs are depleted, corneal regeneration and wound healing are impaired, with the consequent loss of corneal transparency and the impairment of patients’ visual function. The pathology is called LSC deficiency (LSCD), and the loss of transparency is due to a progressive growth of conjunctival cells over the corneal surface, leading to vascularization and corneal opacity associated with chronic inflammation and stromal scarring.

The surgical approaches for this condition required the withdrawal of approximately 40% of limbus from the healthy eye (conjunctival limbal autograft or CLAu) to restore the eyesight of the damaged eye (Keivyon and Tseng, 1989). A proposed alternative for superficial lesions was the simple limbal transplantation, which can induce the re-epithelialization of the treated eye in approximately 6 weeks, as the donor tissue is trimmed and glued over an amniotic membrane (Basu et al., 2016; Magrelli et al., 2020).

To overcome these limitations and predict the quality and success of transplantation, a cell therapy approach including preliminary evaluation of potency, purity, and identity of the treatment, was proposed.

Since epidermal cells from 5 to 7 cm^2^ biopsy could produce as much as 1,8 m^2^ body surface, it was predictable that a very small limbal biopsy (approximately 1–2 mm^2^) could produce one or two corneal surfaces, if cells are isolated and grown in an optimized culture system to obtain a well-differentiated corneal surface (Rama et al., 2010). However, the limbal biopsy was removed at the boundary between the cornea and conjunctiva, with a putative contamination of conjunctival cells that could segregate corneal cells in culture. These conditions require accurate in-process controls to monitor the identity and purity of the cell population. Moreover, LSCD can be treated only by the restoration of stem cells, which should be maintained in sufficient numbers and controlled in culture (the potency of treatment). Stem cells were identified by p63 bright cells, due to strong evidence that their clones (holoclones) had high levels of p63, whereas progenitors expressed low levels of the transcription factor in the nuclei. The knockout mice for p63 confirmed the absence of stratified epithelia.

Indeed, the clinical success of limbal cultures in hundreds of patients correlated to a specific number of transplanted stem cells, described as p63 bright holoclones (Pellegrini et al., 2013). The treatment became a well-established therapy and was acknowledged through conditional approval by the European Medicines Agency in 2015, with the name of Holoclar^®^.

Besides p63, mitotically quiescent limbal stem cells were also characterized by the expression of C/EBPδ and Bmi1 (Barbaro et al., 2007) at molecular level. Other putative stem cell markers such as ATP-binding cassette sub-family B, member 5 (ABCB5) and sub-family G, member 2 (ABCG2) have been described (Sacchetti et al., 2018), but they were never clinically validated.

Currently, numerous attempts at stroma engineering are also ongoing to overcome a deep central cornea replacement by donor tissue, still needed in cases of severe lesions.

Several natural or synthetic corneal stromal substitutes have been proposed for this purpose: collagen polymers, chitosan scaffolds, silk fibroin, hyaluronic acid, and polyarginine are among the most studied biopolymers for corneal wound healing (Mobaraki et al., 2019).

The work in this field is still far from effective routine clinical use.

### Corneal Endothelium: An Example of a Low Regenerative Capacity Tissue

On the internal side of the cornea, another epithelium seals the corneal stroma and is in contact with the aqueous humor, the corneal endothelium (CE). The CE is a simple squamous or cuboidal epithelium, which plays a fundamental role in the maintenance of corneal transparency via dynamic fluid control of its adherent polygonal cells, corneal endothelial cells (CEnCs) (Bourne, 2003).

However, unlike the other epithelia described earlier, CE represents an example of an epithelium with a very low regenerative capacity. Although several markers of putative stem cells (as nestin, p75^*NTR*^, Lgr5, SOX2, p63) have been identified in the CE periphery and some CEnCs have shown a limited ability to replicate (Paull and Whikehart, 2005; McGowan et al., 2007; He et al., 2012; Hirata-Tominaga et al., 2013; Katikireddy et al., 2016; Sie et al., 2020), the majority of them are arrested in G0/G1 phase of cell cycle (Joyce et al., 1996). Growth arrest is associated with a gradual decrease in corneal endothelial cell density, at a rate of approximately 0.6% per year (Bourne, 2003). Whenever CE damage occurs following a physical/surgical trauma or pathology, this is normally compensated by residual CEnC enlargement, migration, and spreading (Tuft and Coster, 1990), confirming the limited CE regenerative capacity *in vivo.* However, since a dysfunctional CE represents one of the major indications for corneal transplantation (Gain et al., 2016), many novel strategies to regenerate this tissue have been proposed and brought to clinic. These approaches include heterologous CEnCs expanded *in vitro* and injected into the patient’s anterior chamber (Okumura et al., 2012; Kinoshita et al., 2018) or implanted through tissue-engineered endothelial keratoplasty (TE-EK) (Peh et al., 2017). A deep knowledge of the finely regulated molecular mechanisms involved in CE proliferation (Joyce, 2012; Frausto et al., 2020; Maurizi et al., 2020) is therefore fundamental to harness a proper approach for this peculiar epithelium, and more advancements are still needed to achieve a full control over its regeneration.

## The Oral Mucosa

An additional epithelium extensively used for regeneration is the oral mucosa. It is composed by a non-cornified squamous epithelium endowed with a great regenerative capacity that is necessary for the reaction to daily damages caused by chewing. Given its easy accessibility and the possibility for multiple biopsies, the oral mucosa is widely used in many surgical reconstructive approaches (Yarington, 1980; Lin et al., 2003; Grimsby and Baker, 2014).

Several studies have been conducted to identify molecular markers for oral mucosa stem cells. Oral mucosa epithelium differs depending on the oral cavity area (buccal, gingiva, hard palate, tongue, etc.), and each study investigated stem cell markers from different oral mucosa sites. It was reported that buccal and gingival cells, expressing high levels of the neurotrophin receptor p75, had greater *in vitro* proliferative capacity and were typically slow cycling *in vivo* (Nakamura et al., 2007). Two additional markers were associated with oral mucosa stem cells, namely p63 (α isoform) and Bmi1 transcription factors. They were enriched in holoclone cells and in young passages of *in vitro* serial cultivation (Corradini et al., 2016).

Many proteins investigated as putative oral mucosa stem cells, were previously reported as skin basal or stem cell markers including integrins, CK 5, 15 and 19, p63, MCSP, CD44H, p75, ABCG2 (Jones and Klein, 2013).

The high regenerative capacity of oral mucosa epithelium has also been investigated, in human and mouse, highlighting an essential role of SOX2 and PITX1 transcriptional networks (Iglesias-Bartolome et al., 2018).

Among the applications of cultured oral mucosa grafts, some were proposed as wound dressings for the treatment of superficial lesion of the oral cavity or chronic ulcers in the digestive tract to promote wound healing.

In the following paragraphs, we are reporting the use of oral mucosal epithelium for engraftment in ophthalmology, where the cultured tissue has shown the same optical transparency of the corneal epithelium (Nishida et al., 2004), and in urology.

### Oral Mucosa Applied for Corneal Epithelial Regeneration in LSCD

In ophthalmology, the oral mucosa epithelium has been used as a corneal substitute in total bilateral LSCD treatment. This pathology is associated with a complete loss of corneal stem cells in both eyes, making autologous therapeutic approaches unfeasible. As an alternative therapy for total bilateral LSCD, allogenic limbal transplantation requires lifelong immunosuppression with systemic complications (Tsai and Tseng, 1994) and results in long-term failures.

To avoid these issues, out of the many autologous cells tested (Homma et al., 2004; Ma et al., 2006; Tanioka et al., 2006; Monteiro et al., 2009; Meyer-Blazejewska et al., 2011; Reza et al., 2011; Yang et al., 2007), only oral mucosal epithelial cells have been successfully applied to humans.

In 2003, Nakamura and co-workers transplanted autologous oral mucosa cells, cultured on an amniotic membrane, in a rabbit LSCD model (Nakamura et al., 2003). The procedure was used in two clinical trials; the first one was applied to six human eyes by the same group (Nakamura et al., 2004) and the second one to four human eyes by a different group (Nishida et al., 2004), using a temperature-responsive support.

These procedures were named “cultivated oral mucosa epithelial transplantation” (COMET) and “cultivated autologous oral mucosal epithelial cell sheet” (CAOMECS) (Burillon et al., 2012), and they have been widely used in the last 20 years in at least 27 published clinical studies (Attico et al., 2021). The reported success rate is approximately 70%, although a comparison between studies is not possible due to the many differences in the diagnosis and the analysis of the results (Cabral et al., 2020).

Indeed, discrepancies were found in LSCD diagnosis and the inclusion criteria of patients affected by different pathologies, making it difficult to understand the impact of treatment on the ailment (Utheim et al., 2016; Cabral et al., 2020; Attico et al., 2021). Moreover, for autologous limbal cells, many different substrates and culture methods have been used for graft preparation. The amniotic membrane (usually de-epithelialized), fibrin gel, and a temperature-sensitive support made by poly(N-isopropyl acrylamide) (Nishida et al., 2004; Satake et al., 2011) have been employed, as also biomaterial-free cultured oral mucosal sheets (Kim et al., 2018).

Additional discrepancies were related to the presence or absence of different types of feeder layers, gamma-irradiated or mitomycin-c-arrested (Kolli et al., 2014; Prabhasawat et al., 2016), to the culture time or to the option of exposing the epithelium to an air-liquid interface stimulus (airlift condition) before transplantation (Cabral et al., 2020).

The behavior of oral mucosal epithelial cells (and so their long-lasting survival) once transplanted remains unclear, and the etiology of LSCD should be considered given that the oral mucosa cell function is strictly dependent on the environment in which they can find different wound beds and signaling.

The *in vivo* behavior of oral mucosal keratinocytes can be evaluated only if the three epithelia that might be present on the ocular surface (ectopic oral mucosa, limbal or conjunctival cells) are univocally distinguished after COMET. To this end, the analyses of the protein markers of the central cornea, frequently removed by keratoplasty following COMET, could provide insights into understanding the regenerative mechanism. Several publications have reported that cultured oral epithelial cell phenotypes, characterized by a panel of common positive and negative proteins, were maintained after successful COMET grafts but not after failures (Nakamura et al., 2007; Chen et al., 2009). Moreover, Soma et al. (2014) demonstrated the survival of GFP-tagged oral cells in a transplanted LSCD rat model, after 8 weeks. Analyses of transplanted rabbit LSCD models rather appeared to contain a mixed epithelium composed of both corneal and mucosal cells on the regenerated *in vivo* tissue (Sugiyama et al., 2014).

The original hypothesis of oral mucosa transdifferentiation (Gaddipati et al., 2014) is no longer considered. It has been proven that epithelia transplanted on an ectopic site of the body retain their differentiated phenotype (Mavilio et al., 2006; Bianco et al., 2013; De Rosa et al., 2014).

In this scenario, the need for a univocal oral mucosal marker is mandatory to unambiguously identify this tissue and to understand the clinical outcome. The current proposals rely only on a panel of non-univocal markers, including mainly cytokeratins, that can be activated *de novo* by different epithelia in pathological environments or in regeneration processes (Moll et al., 2008; Zhang et al., 2019).

Finally, additional studies should also address the topic of angiogenesis related to COMET treatment. Compared to CLET (Cultured Limbal Epithelial Transplant), the epithelium regenerated from the oral mucosa is associated with significant peripheral neovascularization, not impairing the central cornea. In this process, pro-angiogenic and anti-angiogenic factors play different roles (Chen et al., 2012), far to be fully elucidated. Understanding their balance and regulation by the ectopic epithelium could improve the therapeutic protocol for bilateral LSCD.

### The Oral Mucosa to Regenerate Urethra

The oral mucosal epithelium has also been used in urology (Barbagli et al., 2017) for pathologies resulting in a compromised or missing urethral epithelium, which leads to urinary/sexual problems and social discomfort. Hypospadias is one of them and is the most diffused congenital anomaly in men with an incidence rate of 3–7 out of 1,000 new-born babies; it is defined as an incomplete urethral canalization that determines a subsequent fusion of urethral folds during gestation promoted by androgens (Shen et al., 2016).

The human urethra epithelium renews every 3–6 months in physiologic condition (Vaegler et al., 2015). Urothelium turnover suggests the presence of progenitor cells localized in the basal layer, where basal cells express Bmi1 and p63, the latter is one of the main proteins involved in urothelium development and is considered an epithelial stem cell marker (Pechriggl et al., 2013; Gandhi et al., 2014; Corradini et al., 2016). CK5 is exclusively expressed in basal cells as well as CK17, which might be involved in urethral malformations and its role as stem cell marker is still to be understood (Pechriggl et al., 2013). A panel of keratins completed urethral epithelium characterization in the different layers (De Graaf et al., 2017; Sceberras et al., 2020). The first regenerative medicine approach was undertaken in 1990 (Romagnoli et al., 1990): two patients with proximal hypospadias were treated using an epithelium cultivated *in vitro* from autologous urethral cells. Three years later (Romagnoli et al., 1993), a similar approach was used to treat eight hypospadic patients with urethral epithelium cultivated *in vitro* and then mounted on a polytetrafluoroethylene (Gore-Tex) tube.

Later, Atala et al. (1999) used a bladder acellular matrix from cadaveric donors as a scaffold to replace the missing urethra.

Other tissue engineering approaches have been carried out using acellular dermis as a substrate for urothelial cells harvested from bladder washing (Fossum et al., 2012).

None of the many regenerative approaches proposed, however, reached the minimal requirements to become a routine procedure in clinical practice, highlighting the need to further develop the technology.

Currently, the best approaches to treat hypospadias in pediatric patients (Manzoni et al., 2004) rely on several surgical techniques based on the severity of the pathological condition (Xiao et al., 2014). Many of these techniques use a full-thickness oral mucosa graft to replace the missing urethra. The first oral mucosa application for hypospadias treatment was reported by Humby and Higgins (1941). However, the full thickness tissue withdrawal is associated with oral complications such as pain, persistent difficulty in mouth opening, changes in salivary function, and a morbidity rate of 3–4% at donor site (Markiewicz et al., 2008).

To date, the number of recurrences in pediatric patients is significant (Cimador et al., 2013) and is emphasized by the high number of adults suffering from complications due to failed hypospadias treatment (Barbagli et al., 2010).

To reduce the disadvantages of the aforementioned approaches, new products for urethral reconstruction in hypospadias are needed, including standardized, biocompatible, and biodegradable scaffolds. The use of autologous cells, possibly derived from small and non-invasive oral mucosal biopsies, can reduce adverse events (Barbagli and Lazzeri, 2015; Corradini et al., 2016). The maintenance of a sufficient number of stem cells essential for long-term tissue regeneration (Sceberras et al., 2020) and their manufacturing under well-defined culture conditions would improve the clinical outcome. Finally, any new approach should be tested for safety and be able to provide conclusive results for clinical application, including a well-characterized, homogeneous selection of patients.

In a different urological disorder, Ram-Liebig and colleagues in 2015 provided an interesting example of a new tissue-engineered oral mucosa graft (Ram-Liebig et al., 2015). A small autologous oral mucosa biopsy was obtained from the patients, the extracted cells were cultured on a biocompatible scaffold and applied to patients (Ram-Liebig et al., 2017). The product, approved in Germany and commercialized as MukoCell^®^, showed a significant efficacy (84%) in treated patients (Barbagli et al., 2018).

## Salivary Glands

The salivary glands (SGs), anatomically connected with the oral mucosa, are essential structures responsible for saliva production. This fluid is mainly involved in fundamental functions such as digestion, regeneration of oral and esophageal mucosa, and protection from bacterial infection and dental caries (Khan et al., 2020). Many pathological conditions such as infections, autoimmune diseases (e.g., Sjogren’s syndrome), metabolic disorders or consequences of radiation therapy (RT) targeting head and neck cancers can drastically damage SGs, with severe repercussions for patients’ quality of life. Until now, no determined therapeutic approaches are available to treat salivary dysfunctions like xerostomia (dry mouth syndrome); hence current treatments are mainly symptomatic. For this reason, there are many ongoing studies aimed at developing novel regenerative strategies.

The SG presents several progenitor populations that are able to regenerate the tissue, depending on the extent and the location of the damage (Porcheri and Mitsiadis, 2019), as highlighted by studies mainly based on animal models. Among the many putative molecular markers investigated to identify the SG stem-progenitor cells, c-kit, SOX2, CK5 and ASCL3 seem to have a major role. C-kit + cells can proliferate and differentiate *in vivo* and *in vitro* and, more importantly, to restore the salivary secretion when transplanted in murine irradiated SGs (Lombaert et al., 2008a). Analogously, the expression of the transcription factor ASCL3 seems to regenerate both acinar and ductal cells (Bullard et al., 2008). However, when ASCL3 is ablated, the SG appear smaller than the control but retain the presence of a population of CK-5 + basal progenitor cells, highlighting a compensatory mechanism among different stem-progenitor populations (Arany et al., 2011). Finally, a subset of SOX2-expressing acinar cells (SG secreting cells) was found to replace acinar cells after SG irradiation through a SOX2 nerve-dependent mechanism (Emmerson et al., 2018).

Currently, the regenerative strategies aimed at restoring the SG function include cell-based therapy, gene-therapy and bioengineering strategies. Among cell-based therapies, starting from 2008, Coppes’s group demonstrated the ability of rodent SG-specific epithelial cell transplantation to restore SG after RT (Lombaert et al., 2008a; Nanduri et al., 2013). These cells can be safely cryopreserved for an extended period (Nanduri et al., 2013), and their stem cell pool can be enriched (Nanduri et al., 2013). Preliminary results in humans, derived from the isolation and expansion of SG stem-progenitor cells, would suggest a similar restoring ability. However, further studies are needed to verify the clinical applicability of these stem-progenitor cells (Feng et al., 2009; Pringle et al., 2016).

Different cell therapy approaches involve the use of non-epithelial cell types to activate regenerative mechanisms on residual SG after RT or recreate the surrounding environment. Among them, human adipose-derived mesenchymal stem cells (hAdMSCs) have shown to reduce cell apoptosis and tissue fibrosis and differentiate in SG endothelial cells (Kojima et al., 2011; Lim et al., 2013). Moreover, in 2017, the first in man double-blinded, Phase I/II Clinical Trial was performed to evaluate safety and feasibility of this type of cells to treat radiation-induced xerostomia (no results yet) (Grønhøj et al., 2017). Similarly, Bone Marrow (BM)-derived cells were found to increase microvessel density and enhance saliva secretions by inducing epithelial repair (Lombaert et al., 2008b).

Besides cell-based therapy, promising results came from gene therapy with adenoviral vectors to deliver hAQP1 and KGF genes. Notably, the administration of hAQP1 gene in irradiated parotid glands led to an increase in saliva production in 5 out of 11 treated patients, who maintained stable results up to 5 years (Baum et al., 2012; Alevizos et al., 2017). Instead, the administration of KGF genes in mice before irradiation prevents salivary hypofunction with no effect on tumor growth, suggesting a putative future clinical application (Zheng et al., 2011).

Finally, bioengineering and TE strategies are under evaluation for the regeneration of damaged SGs. Despite a plethora of different substrates tested, the optimal scaffold for *in vivo* SG regeneration has not yet been identified (Mitroulia et al., 2019). Nevertheless, a promising proof-of-concept for regenerative organ replacement comes from bioengineering, as in 2013, Ogawa and colleagues demonstrated the regeneration of an utterly functional SG through the orthotopic implantation in mice, of a bioengineered SG germ. The bioengineered graft went through successful morphogenesis; it perfectly integrated with the recipient salivary duct and exerted the main SG functions (Ogawa et al., 2013).

In conclusion, until now, no SG regeneration strategies have yet become a real therapeutic alternative for the patient suffering from xerostomia, despite the progress obtained in the last years. Since most of the studies are based on animal models, further work is needed to validate the results in humans.

## The Airways

In contrast to the previously described epidermal, corneal and mucosal human epithelia, an effective approach to reconstruct the airway epithelium has not yet been clinically established. Some of the problems that limit airway regeneration include difficulties observed by several groups to effectively grow primary human respiratory epithelial cells (Widdicombe et al., 2005; Walters et al., 2013; Butler et al., 2016) to maintain the stem cell pool and their potential to regenerate all specialized cell types, as well as the reconstruction of a long, full-thickness respiratory tract.

In addition, the airway structure and its epithelium drastically change from the nasal cavities to the alveoli, and different stem/progenitor cells populations have been proposed for the various respiratory tracts, mainly relying on lineage tracing experiments and airway injury models (Basil et al., 2020; Parekh et al., 2020). The basal cells, characterized by the expression of CK5 and CK14 and the transcription factor p63, have been identified as the primary multipotent stem cells population of the tracheobronchial pseudostratified epithelium due to their capacity to self-renew and differentiate into the supra-basal cell types (Rock et al., 2009; Rock et al., 2010). Deep into distal airways, SCGB1A1 + club cells act as progenitors for the goblet and ciliated cells (Zuo et al., 2018; Parekh et al., 2020). Following an injury, these cells can also dedifferentiate acquiring a basal cell morphology and regenerate alveolar type I and II cells (Barkauskas et al., 2013; Tata et al., 2013). Meanwhile, the regeneration of the alveolar epithelium depends on the presence of cuboidal alveolar type II SFTPC + cells (AT2) which can self-renew and give rise to the gas-exchanging AT1 cells (Barkauskas et al., 2013) other than secrete surfactants proteins.

The complexity of the several compartments of the respiratory system highlights the necessity to consider a specific reconstructive approach for each district.

A wide range of approaches has been tested for tracheal and main bronchi reconstructions. The clinical use of synthetic prosthesis is characterized by high morbidity and mortality, so that solid substitutes are no longer considered suitable for long-term tracheal replacement, and the use of porous prosthesis is limited to laryngeal replacement (Etienne et al., 2018).

Decellularized aortic and tracheal allografts are biocompatible and do not require immunosuppression, although their clinical applications are characterized by a high number of re-interventions and high morbidity. Indeed, the absence of a continuous respiratory epithelium and poor mechanical properties require stent application with increased risks of chronic inflammation, infection, and damage to the surrounding tissues (Etienne et al., 2018).

With regard to the tissue engineering strategies, in 2008, the first bioengineered trachea was implanted in a patient affected by severe bronchomalacia (Macchiarini et al., 2008), and despite the complications, other subjects were treated with a similar therapeutic approach (Hamilton et al., 2015). However, morbidity and a high rate of lethality were observed during the follow-up period (Gonfiotti et al., 2014; Elliott et al., 2017). The recurrence of stenosis, infections, and inflammation within the transplanted bioengineered constructs was- linked to the lack of correct vascularization and epithelialization (Pepper et al., 2019; Niermeyer et al., 2020). Previous studies have highlighted the need for appropriate preclinical data to optimize the scaffold, for the identification and culture of all cell types to manufacture grafts, and the lack of studies on the interactions between cells and cell-scaffolds (Niermeyer et al., 2020).

## The Intestine

The inner epithelium of the human intestine, with a surface of > 30m^2^, is the second-largest tissue of our body (Gehart and Clevers, 2019). It is involved simultaneously in two main functions: metabolites absorption and barrier protection against potentially noxious microorganisms or environmental insults. It undergoes continuous mechanical stress that causes the death of 1011 intestinal epithelial cells every day (Barker, 2014): the life-time of a mature intestinal epithelial cell is therefore very short (3–5 days) (Darwich et al., 2014). This elevated cell loss is compensated by a high self-renewal rate, triggered by a stem cell population residing within invaginations of the intestinal epithelial wall, the crypts. The crypt is composed of continuously dividing crypt base columnar cells (CBCs), interspaced with Paneth cells, an essential source of niche factors. The CBCs have been identified through several markers, including Lgr5, Ascl2, Prom1, Olfm4 and Smoc2 (Kim et al., 2017). In addition to the CBC, another population of quiescent reserve stem cells has been identified at position + 4 from the bottom of the crypt and is characterized by Bmi1, Tert, Hopx and Lrig1 (Gehart and Clevers, 2019). The stem cells give rise to the transient amplifying cells that rapidly divide and become mature, while being pushed out from the crypt to reach the villus’ tip, where they eventually undergo apoptosis, exfoliating into the lumen. Six types of mature intestinal cells can be distinguished in absorptive (enterocytes and M cell) and secretory (goblet, Paneth, enteroendocrine and tuft cell, the latter involved in immune regulation).

The high regenerative capacity of this epithelium, with stem cells able to self-renew constantly throughout life, makes this tissue ideal for regenerative medicine approaches. At the same time, however, the continuous proliferation of intestinal stem cells accumulates mutations that can promote cancer growth. The most effective treatments for cancers remain nowadays radiotherapy and chemotherapy, even though they induce severe damages to the normal tissues and their side effects are more evident in rapidly renewing tissues such as hematopoietic system and gastrointestinal tract (Yu, 2013). Lgr5+ CBC are particularly susceptible to radiation, and they are replenished by reserve stem cells, which appears to be radioresistant given their quiescence (Kim et al., 2017). Although intestinal cells, following irradiation, showed high plasticity in regenerating the epithelium, the administration of mesenchymal stem cells demonstrated a reduction in the inflammatory response, facilitating epithelial regeneration (Qi et al., 2020).

The intestine is very difficult to regenerate due to its complex structure, pH conditions and its enteric nervous associated motility. Use of decellularised extracellular matrix scaffolds shows promises in regenerating the intestinal trait in preclinical studies, even though they promoted substantial contractions upon implantation (Hussey et al., 2017).

The breakthrough in intestinal regeneration has been developing intestinal organoids (Sato et al., 2009). Lgr5+ cells in organoids guarantee the self-renewal of these “mini-guts,” which can exert both digestive and absorptive functions. Intestinal organoids have been used to study cancer progression and mutagenesis (Drost et al., 2015; van de Wetering et al., 2015; Fumagalli et al., 2017) and study CFTR function in cystic fibrosis (Dekkers et al., 2016). A recent study demonstrated for the first time the success obtained following transplantation of human colon organoids into receiving mice (Sugimoto et al., 2018). Human intestinal organoids could be instrumental for developing novel approaches in regenerative medicine with the aid of a phenotypic landscape (Shin et al., 2019; Lukonin et al., 2020) to understand signaling pathway and biomaterials to favor implantation.

## Discussion

The number of attempts involving epithelial regeneration clearly shows that these tissues’ absence represents a huge medical problem (Kucharzewski et al., 2019; Table 1). Even a partial disruption of the epithelial tissues, the most common being a penetrating infection, dramatically increases the likelihood of health impairment and illustrates the importance of epithelia in body functions.

**TABLE 1:** Summary of published clinical applications involving autologous cultured epithelia engraftment.

Epithelium regeneration from	Condition	Total patients treated	Follow up (years)	Countries involved	First clinical application	Differences related to
Skin	Burns	< 1000	< 5	US, EU, Japan and others	O’Connor et al., 1981	
	Vitiligo	> 100	< 5	US, EU, India, Turkey	Falabella et al., 1989	
	JEB	< 10	> 5	EU	Mavilio et al., 2006	
Cornea	LSCD	< 1000	∼ 10	EU, Japan, US, India, Taiwan, Iran	Pellegrini et al., 1997	
Oral Mucosa	LSCD	> 100	> 10	Japan, EU, Taiwan, India, Thailand, Iran, South Korea, China, Malaysia	Nakamura et al., 2004	
	Urethral Strictures	∼ 100	< 5	EU	Ram-Liebig et al., 2015	
Airways	Stenosis, cancer	< 10	∼ 5	EU	Macchiarini et al., 2008	
						Carriers, feeder layers, cell source, serum, hormones, growth factors, culture medium, air lifting, matrices, cell types

*JEB, junctional epidermolysis bullosa; LSCD, limbal stem cells deficiency.*

It is worth noting that humans can live after partial heart impairment, with limited pancreatic activity or partial brain loss, but not with a partial absence of epithelia in any part of the body. As signaling and secretory tissues, these barriers are associated with a critical immunosurveillance, making the therapeutic use of allogenic tissues, only a temporary solution under immunosuppression or a wound dressing.

The cornea has been considered a typical exception, as an immune-privileged site. However, this is not entirely true (Henderson et al., 2001; Inomata et al., 2020). Indeed, human corneal endothelial cells are a potential target of immune attack after corneal transplantation, and the limbus, containing corneal stem and progenitor cells, are vascularized and immunoreactive to allogeneic components (Lahdou et al., 2013).

The importance of immunological reactions can be related to the use of allogeneic or xenogenic cells from some matrix sources, as well as to the inflammatory reactions driven by some materials used for tissue regeneration. This was the case with tracheal and many urethral or intestine scaffolds, which produced significant adverse reactions in *in vivo* studies.

The introduction of a fully biocompatible support, such as fibrin glue or amniotic membrane, resulted in a significant improvement of different processes: avoided the shrinking of the epithelium, thus enabling the coverage of large wound surfaces in a short time, in addition to safe long-distance transport.

**FIGURE 2:**
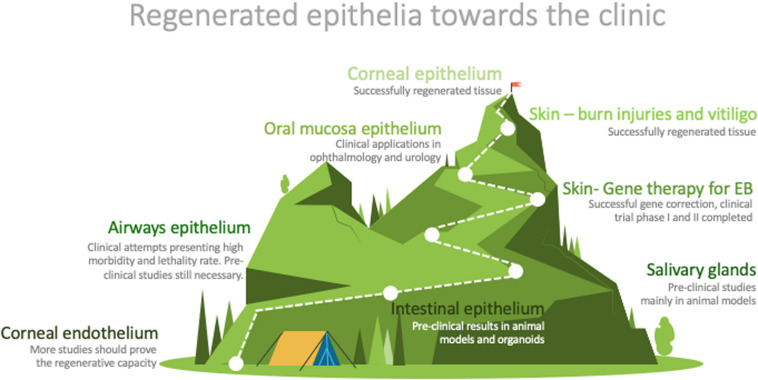
Graphic representation of the distance between research and routine clinical application of the described epithelia. Designed by PoweredTemplate.

The easy access to some epithelia does not guarantee the development of successful clinical applications (Figure 2). However, the accessibility to epithelia has been the starting point for much research in the field. The knowledge achieved has laid the groundwork for some very effective treatments for several pathologies, such as burns, skin pigmentation disorders, ocular defects, and some genetic conditions (Figure 2).

Additional progress will come from multidisciplinary interactions, providing different perspectives and different solutions supporting treatments’ efficiency. Examples can be the development of alternative diagnostic methods aimed at the precise grading of some pathologies, the pharmaco-toxicology of drugs on cultured tissues or in the whole treatment, the functionalization/patterning of surfaces by nanotechnology, and the analysis of predictive markers for responses to the therapeutic interventions.

Many conclusions can be drawn from these different applications: altogether, these experiences suggest several improvements for the therapeutic use of advanced therapy medicinal products. Without being exhaustive, the list of problems includes the need for standardization, which is common to most therapies. The non-homogeneous selection of pathologies included in the preliminary clinical trials produced significant intra- and inter-pathology heterogeneity, associated with differences in the corresponding microenvironments and significantly contributed to variable cell behavior. Within these microenvironments, the extracellular matrix and paracrine signaling can profoundly influence the way cells engraft, grow, differentiate, or persist.

Additional common issues were the incomplete cell characterization of the cultured tissues and some lack of comparison with their *in vivo* counterparts, frequently impairing the clinical outcome and related evaluations. Therefore, a more exhaustive cell characterization, together with specific stem cell identification, are instrumental for self-renewal and long-term tissue maintenance.

The nature of stem cells could change under homeostatic conditions or during tissue repair. The essential characteristics of the stem cells, like the specific location and marker expression, quiescence, asymmetric division, and unidirectional differentiation, do not apply to all tissues, as shown in the airways. In a pragmatic vision, the focus on the search for stem cells should move from their physical identification to their function, meaning the tissue ability to restore all cell types, over the life-time. Preclinical and clinical studies must support the validation of the selected markers for their role in these adult stem cells’ structural and functional regeneration capabilities.

The standardization of methods does not mean having one single procedure; rather, it means that scientific experts would define the available golden standard time by time, by selecting the most characterized and safe method from a scientific and regulatory perspective.

This approach would enable bridging new methods to the golden standard, as the most characterized procedure, to highlight different cell/tissue behavior and define the best risk/benefit ratio. Agreements on methods and controls should also be based on long-term follow-up data of previous treatments, which implies a shift from estimating probabilities to relying on certainties, without censuring the possibility of medical innovation (Sugarman et al., 2018). The World Medical Association in the declaration of Helsinki, literally reported: “in the treatment of an individual patient, where proven interventions do not exist, or other known interventions have been ineffective, the physician, after seeking expert advice, with informed consent from the patient or a legally authorized representative, may use an unproven intervention if, in the physician’s judgment, it offers hope of saving life, re-establishing health, or alleviating suffering.” This intervention should subsequently be made the object of research, designed to evaluate its safety and efficacy. In all cases, new information must be recorded and, where appropriate, made publicly available (The World Medical Association Inc, 2008).

## Author Contributions

EM, DA, FM, GG, EA, AM, BM, VG, VS, and GP contributed to the analysis of literature, writing and correction, and figures. LL contributed to the analysis of literature, revision of the manuscript, and figures. EM and DA collected and summarized the whole work. GP also provided the final revision. All authors contributed to the article and approved the submitted version.

## Conflict of Interest

EM, FM, AM, VG, VS, and GP are employees of Holostem Terapie Avanzate, producing an Advanced Therapy Medicinal Product for corneal restoration. The remaining authors declare that the research was conducted in the absence of any commercial or financial relationships that could be construed as a potential conflict of interest.
